# Efficacy of intravascular imaging-guided drug-eluting stent implantation: a systematic review and meta-analysis of randomized clinical trials

**DOI:** 10.1186/s12872-022-02772-w

**Published:** 2022-07-23

**Authors:** Ying Niu, Nan Bai, Ying Ma, Peng-Yu Zhong, Yao-Sheng Shang, Zhi-Lu Wang

**Affiliations:** 1 The First Clinical Medical College of Lanzhou University, Lanzhou, China; 2 Department of Cardiology, The First Hospital of Lanzhou University, No. 1, Donggang West Road, Chengguan District, Lanzhou, 730000 Gansu China

**Keywords:** Intravascular imaging, Intravascular ultrasound, Optical coherence tomography, DES, Meta-analysis

## Abstract

**Background:**

Traditional angiography only displays two-dimensional images of the coronary arteries during stent implantation. However, intravascular imaging can show the structure of the vascular wall, plaque characteristics. This article aims to evaluate the efficacy of intravascular imaging-guided drug-eluting stent (DES) implantation.

**Method:**

We conducted a systematic review and meta-analysis of randomized controlled trials of intravascular imaging-guided, including patients with DES implantation guided by intravascular ultrasound or optical coherence tomography and traditional angiography. The databases of PubMed, EMBASE, web of science, and Cochrane Library were searched. The primary outcome was target lesion revascularization (TLR). The secondary outcomes included the target vessel revascularization (TVR), myocardial infarction (MI), stent thrombosis (ST), cardiac death, all-cause death, and the major adverse cardiac events (MACE) during the 6–24 months follow-up. The fixed-effects model was used to calculate the relative risk (RR) and 95% confidence interval of the outcome event. Meanwhile, the trial sequence analysis was employed to evaluate the results.

**Result:**

This meta-analysis included fourteen randomized controlled trials with 7307 patients. Compared with angiography-guided, intravascular imaging-guided DES implantation can significantly reduce the risk of TLR (RR 0.63, 0.49–0.82, *P* = 0.0004), TVR (RR 0.66, 0.52–0.85, *P* = 0.001), cardiac death (RR 0.58; 0.38–0.89; *P* = 0.01), MACE (RR 0.67, 0.57–0.79; *P* < 0.00001) and ST (RR 0.43, 0.24–0.78; *P* = 0.005). While there was no significant difference regarding MI (RR 0.77, 0.57–1.05, *P* = 0.10) and all-cause death (RR 0.87, 0.58–1.30, *P* = 0.50).

**Conclusions:**

Compared with angiography, intravascular imaging-guided DES implantation is associated with better clinical outcomes in patients with coronary artery disease, especially complex lesions (Registered by PROSPERO, CRD 42021289205).

**Supplementary Information:**

The online version contains supplementary material available at 10.1186/s12872-022-02772-w.

## Introduction

Cardiovascular disease remains the most common cause of death in the world, and its prevalence is constantly increasing [1]. Coronary atherosclerosis is one of the main causes of cardiovascular disease. For quite some time, coronary angiography is considered the “gold standard” for diagnosing coronary artery disease and remains the main imaging modality used worldwide for vascular imaging, and percutaneous coronary intervention guided by angiography is the main revascularization strategy for patients with coronary artery disease [2, 3].

However, angiography underestimates the true vessel size, lesion length, and degree of calcification, and cannot further evaluate plaque morphology, plaque vulnerability, presence of thrombus, stent expansion and apposition, residual narrowing post intervention, and the presence of dissections [2]. On the contrary, intravascular imaging can provide more detailed information of vascular lumen and wall to guide the intervention therapy. Therefore, intravascular imaging including intravascular ultrasound (IVUS) and optical coherence tomography (OCT) is more and more widely used in the percutaneous coronary intervention compared with angiography [4]. Meanwhile, the 2011 American College of Cardiology Foundation/American Heart Association/Society of Cardiovascular Angiography and Intervention Guideline for Percutaneous Coronary Intervention recommends that IVUS may be considered for the guidance of left main coronary artery stenting (IIb) [5]. Although 2018 European Society of Cardiology guidelines on myocardial revascularization recommend IVUS to guide stent implantation for left main coronary artery lesions (IIa), this recommendation is based on a multicenter registry study [6]. In addition, many randomized trials and observational studies have shown that the beneficiaries are not limited to patients with left main coronary artery lesions [7–9]. Therefore, whether intravascular imaging has clinical benefits remains unclear in all patients undergoing percutaneous coronary intervention, regardless of the type of lesion.

We performed a meta-analysis of randomized controlled trials comparing intravascular imaging-guided and angiography-guided stent implantation, to explore the efficacy in patients with coronary artery disease receiving percutaneous coronary intervention. The results indicate that stent implantation guided by intravascular imaging is more effective in patients with coronary artery disease, and complex lesions benefit more.

## Method

### Data source, search strategy and quality assessment

This systematic review and meta-analysis of randomized controlled trials followed the Preferred Reporting Items for Systematic Review and Meta-Analysis (PRISMA) guideline [10]. We searched PubMed, Web of Science, EMBASE, and Cochrane Library databases from inception to 13, April 2022, and the following search terms and keywords were used: “angiography”, “angiography-guided”, “intravascular ultrasound”, “intravascular ultrasound-guided”, “IVUS”, “optical coherence tomography”, “optical coherence tomography-guided”, “OCT”, “stent implantation”, “percutaneous coronary intervention”, “PCI”. There were no language restrictions for retrieval. The search strategy of each database is shown (Additional file 2: Table S1). The inclusion criteria of this study: (a) randomized controlled trial; (b) comparison between coronary drug-eluting stent (DES) implantation guided by IVUS or OCT and angiography-guided; (c) follow-up for at least 6 months; (d) sample size > 100 patients; (e) availability of complete clinical and outcome data. The exclusion criteria of this study: (a) ongoing trials and non-randomized controlled trials; (b) trials did not have the outcomes needed or the data of incomplete outcomes; (c) provisional stenting strategy; (d) meta-analyzes, reviews, or comments. In this meta-analysis, two investigators (Ying Niu and Nan Bai) independently screened all titles and abstracts, full-text articles of relevant trials, and then evaluated the eligibility of the trials following the inclusion and exclusion criteria. The disagreement was discussed to resolve by a third party (Ying Ma, Peng-Yu Zhong, and Yao-Sheng Shang). The risk of bias for each trial was assessed by the Cochrane tool of collaboration, and the quality of evidence for each outcome was evaluated by the Grades of Recommendations Assessment Development and Evaluation (GRADE) [11, 12]. The clinical protocols of all included trials were approved by local ethics and informed consent of patients was obtained. Meanwhile, this study is a secondary research and does not require ethical approval, and the meta-analysis protocol was registered in PROSPERO (CRD 42,020,289,205).

### Data acquisition and clinical outcomes

The two investigators independently extracted the characteristics of each trial included, the baseline characteristics of the patients, and the outcome of each trial. The differences should be settled by a third party through consultation (Zhi-Lu Wang). The primary outcome was target lesion revascularization (TLR). The secondary outcomes included target vessel revascularization (TVR), myocardial infarction (MI), cardiac death, all-cause death, stent thrombosis (ST), and major adverse cardiac events (MACE). MACE was defined as the composite of all-cause death or cardiac death, MI, and repeat coronary revascularization. The latter was assumed as TLR, TVR, or any coronary revascularization. TLR, MI, cardiac death, all-cause death, and ST was defined based on the definition adopted of the clinical trials included. Meanwhile, based on the trials included, complex lesions were defined as one of the following: lesion type B2 and C according to the American Heart Association; chronic total occlusions (CTO); bifurcation lesions; proximal left anterior descending artery; long lesions (> 20 mm); small vessels (reference vessel diameter ≤ 2.5 mm); left main coronary artery lesions and patients requiring 4 or more stents; insulin dependent diabetes mellitus and acute coronary syndrome.

### Statistical analysis

All data were analyzed by Review Manager version 5.4 software (The Nordic Cochrane Center, Copenhagen, Denmark) and Stata version 14.0 software. The risk ratio (RR) and 95% confidence interval (CI) of each outcome were expressed and calculated by the fixed-effects model and Mantel–Haenszel method, and the statistical heterogeneity between trials was assessed with chi-square tests and *I*^*2*^ statistics. When the *P-*value of the within-group heterogeneity chi-square test was < 0.10, between-group heterogeneity chi-square test was < 0.05, significant heterogeneity was considered, and *I*^*2*^ was used to judge the degree of heterogeneity. The sources of heterogeneity were found through sensitivity analysis and subgroup analysis. Meanwhile, the sensitivity analysis was employed to test the impact of any individual study results on the overall results. Egger’s and Bgge’s test as well as visual inspection of funnel plots were used to assess publication bias, and the trim method will be used when the Egger’s test *P* < 0.05. Finally, calculate the sample size followed by Trial Sequential Analysis version 0.9.5.10 software (Copenhagen Trial Unit, CTU) and evaluate the results.

## Results

### Search results and study characteristics

**Fig. 1:**
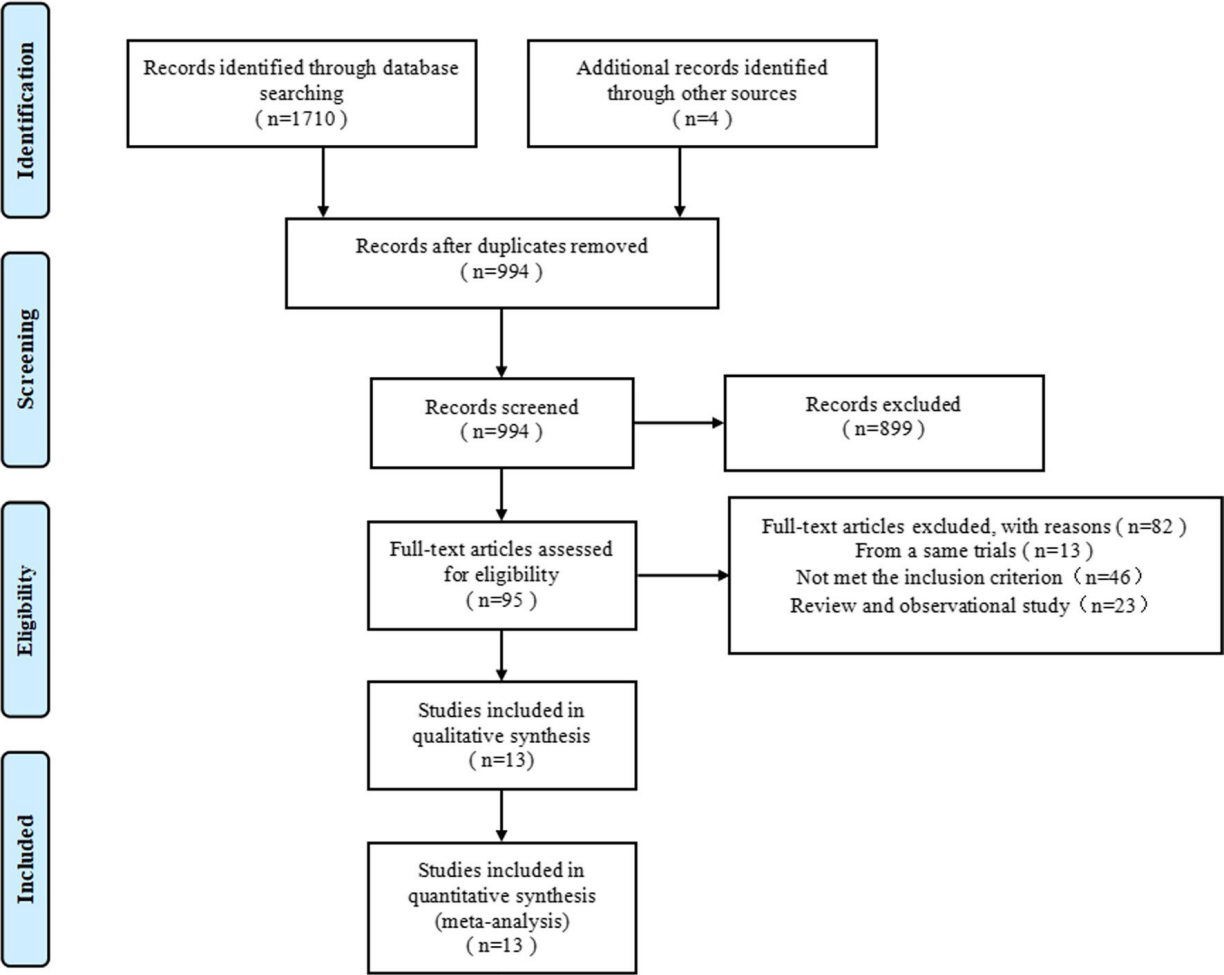
Flow diagram of literature search

A total of 1714 articles were retrieved, and 994 citations were screened by checking the title or abstract. Of these, 95 full texts were reviewed, and thirteen randomized controlled trials were included in this meta-analysis finally (Fig. 1).

The baseline characteristics of the included trials were shown (Table 1). A total of 7307 patients were selected, including 3659 patients receiving intravascular imaging-guided stent implantation and 3648 patients receiving angiography-guided stent implantation. The enrolled population of ten trials was patients with complex lesions [13–20, 24], two trials included patients with left main coronary artery lesion [18, 19], and five trials excluded obvious left main coronary artery lesion [15, 17, 21, 22]. Meanwhile, the outcomes of subgroup for these patients were also reported. One trial was related to OCT vs angiography [22], ten trials were related to IVUS vs angiography [13–20, 23, 24], and two trials were related to OCT vs IVUS vs angiography [21, 34]. Two trials included patients with new-generation DES [15, 16]. The follow-up time ranged from nine months to three years. In addition, all trials reported the outcome of MACE and we showed the difference defined of MACE in Table 1.

**Table 1:** Baseline characteristics of the included trials

Study	Publication year	Type	Country	Lesion type	DES type	Study total size	Randomization	MACE	Follow up (month)
AIR-CTO [13]	2015	RCT	China	CTO	First/Second generation	115/115	IVUS VS Angiography	All-cause death, MI, TLR, ST	24
AVIO [14]	2013	RCT	European countries	Complex lesions^a^	First generation	142/142	IVUS VS Angiography	Cardiac death, MI or TVR	24
CTO-IVUS [15]	2015	RCT	Korea, America	CTO	New-generation	201/201	IVUS VS Angiography	Cardiac death, MI or TVR	12
HOME DES IVUS [23]	2010	RCT	Czech Republic	Complex lesions^a^	First generation	105/105	IVUS VS Angiography	All-cause death, MI or TLR	18
ILUMIEN III [21]	2021	RCT	America	Non-complex lesions	NR	289/142	IVUS + OCT VS Angiography	Cardiac death, MI or TLR	12
iSIGHT [34]	2021	RCT	Brazil	Long lesions, Non–unprotected left main	Second/ New-generation	101/49	IVUS + OCT VS Angiography	Cardiac death, MI and TLR	30
IVUS-XPL [16]	2015	RCT	Korea	Long lesions	New-generation	700/700	IVUS VS Angiography	Cardiac death, MI or TLR	12
Kala et al. [22]	2016	RCT	Czech Republic, America	All comer	Second/ New-generation	105/96	OCT VS Angiography	All-cause death, MI, and TLR	9
Kim et al. [17]	2013	RCT	Korea	Long lesions	Second generation	297/246	IVUS VS Angiography	Cardiac death, MI, TVR or ST	12
Liu et al. [18]	2019	RCT	China	Unprotected left main	NR	167/169	IVUS VS Angiography	Cardiac death, MI or TVR	12
RESET [24]	2013	RCT	Korea	Non-complex lesions	Second generation	662/912	IVUS VS Angiography	Cardiac death, MI or TVR	12
Tan et al. [19]	2015	RCT	China	Unprotected left main	First generation	61/62	IVUS VS Angiography	All-cause death, MI, TLR	24
ULTIMATE [20]	2021	RCT	China	Long lesions	Second generation	714/709	IVUS VS Angiography	Cardiac death, MI or TVR	36

*RCT* randomized controlled trial; *CTO* chronic total occlusion; *IVUS* intravascular ultrasound; *OCT* optical coherence tomography; *DES* drug-eluting stent; *MI* myocardial infarction; *TLR* target lesion revascularization; *ST* stent thrombosis; *TVR* target vessel revascularization; *RCR* repeat coronary revascularization; *NR* not reported

^a^Based on the trials included, complex lesions were defined as one of the following: lesion type B2 and C according to the American Heart Association; chronic total occlusions (CTO); bifurcation lesions; proximal left anterior descending artery; long lesions (> 20 mm); small vessels (reference vessel diameter ≤ 2.5 mm); left main coronary artery lesions and patients requiring 4 or more stents; insulin dependent diabetes mellitus and acute coronary syndrome

The baseline clinical characteristics of the included patients were shown (Table 2). In all trials included, the average age of patients was approximately 64 years old in the intervascular imaging-guided coronary stenting group and about 67.9% of patients were male. In addition, 29.9% of patients had diabetes, 54.1% of patients suffered from dyslipidemia, 64.5% of patients accompanied hypertension, and 27.9% of patients had a history of current smoking. The period of follow-up ranged from 9 to 36 months. Meanwhile, the average age of patients was approximately 64 years old in the angiography-guided coronary stent implantation, of which 69.4% of patients were male. Furthermore, 30.6% of patients had diabetes, 55.2% of patients merged dyslipidemia, 63.2% of patients amalgamated hypertension, and 32.2% of patients suffered from a history of current smoking approximately. Angiography and procedural characteristics are shown (Table 3).

**Table 2:** Baseline clinical characteristics of the included patients

	AIR-CTO [13]	AVIO [14]	CTO-IVUS [15]	HOME DES IVUS [23]	ILUMIEN III [21]	iSIGHT [34]	IVUS-XPL [16]	Kala et al. [22]	Kim et al. [17]	Liu et al. [18]	RESET [24]	Tan et al. [19]	ULTIMATE [20]
Patients (n)	115/115	142/142	201/201	105/105	136/153/142	50/51/49	700/700	105/96	269/274	167/169	662/912	61/62	724/724
Age (mean)	67/66	64/64	61/61	59/60	66/66/67	59/60/59	64/64	57/59	63/64	65/65	61/63	77/76	65/66
Male (%)	89/80	82/77	81/81	73/71	74/69/73	36/31/38	69/69	83/87	66/55	64/64	65/65	62/69	74/73
Current smokers (%)	39/39	35/31	35/34	40/35	13/17/23	14/17/14	22/26	64/59	22/17	37/36	25/26	44/47	35/32
Hypertension (%)	75/70	70/67	63/64	67/71	78/78/75	42/46/39	65/63	50/52	61/66	70/72	60/63	41/47	71/72
Dyslipidemia (%)	22/28	70/77	NR	63/66	75/73/77	30/36/28	67/65	NR	61/62	38/38	61/56	NR	54/55
Diabetes (%)	30/27	24/27	35/34	42/45	36/33/28	20/17/22	36/37	17/26	32/30	34/31	28/30	34/30	30/31
Previous MI (%)	21/30	NR	8/8	37/32	NR	32/29/28	5/4	1/6	1/3	17/14	1/2	16/21	9/12
Previous PCI (%)	NR	NR	15/16	17/14	NR	NR	11/10	4/4	NR	20/17	3/3	NR	17/20
Previous CABG (%)	NR	NR	2/3	14/10	NR	NR	3/2	0/0	NR	1/1	NR	NR	1/1
LVEF (%)	55/56	55/56	57/57	NR	NR	NR	63/62	NR	55/54	56/58	NR	55/53	61/60
Unstable angina (%)	9/10	30/26	NR	43/39	NR	NR	35/32	NR	38/39	76/75	NR	70/66	67/64
Stable angina (%)	71/76	70/64	100/100	38/40	35/34/35	18/22/21	51/51	NR	53/52	12/11	40/45	30/34	13/13
ACS (%)	29/24	30/26	NR	72.60	36/33/36	NR	49/49	NR	47/49	86/87	45/55	NR	79/78

*MI* myocardial infarction; *PCI* percutaneous coronary intervention; *CABG* coronary artery bypass grafting; *LVEF* left ventricular ejection fraction; *ACS* acute coronary syndrome; *NR* not reported

**Table 3:** Angiographic and procedural characteristics of the included patients

	AIR-CTO [13]	AVIO [14]	CTO-IVUS [15]	HOME DES IVUS [23]	ILUMIEN III [21]	iSIGHT [34]	IVUS-XPL [16]	Kala et al. [22]	Kim et al. [17]	Liu et al. [18]	RESET [24]	Tan et al. [19]	ULTIMATE [20]
Stent diameter (mm)	3.1/2.9	3.0/2.9	2.9/2.9	NR	NR	3.3/3.3/3.2	NR	NR	NR	3.5/3.3	NR	3.4/3.4	3.1/3.0
Stent length (mm)	55/52	23.9/23.2	43.6/41.5	23.6/22.1	24/23/20	32.5/28.6/25.8	39.3/39.2	NR	33/31	32.6/33.3	20.4/20.1	21.5/18.2	50.0/47.4
Max balloon diameter (mm)	NR	3.4/3.2	NR	3.3/3.1	3.5/3.5/3.0	3.5/3.5/3.5	3.1/3.0	NR	3.2/3.1	3.5/3.5	NR	NR	3.7/3.5
Max post-dilation pressure (Atm)	NR	20.3/19.6	14.6/13.8	16.4/15.2	19/18/18	20/20/24	16.5/15.9	18/16	13.4/13.6	15.4/13.9	16.7/16.1	NR	19.7/19.0
Contrast volume (ml)	293/293	NR	299/295	133/113	196/225/183	8NR	NR	230/168	NR	NR	NR	NR	178/162
Lesion length (mm)	29.0/30.6	27.4/25.5	36.3/35.5	18.1/17.6	15.3/15.3/14.7	23.1/21/6/20.2	34.7/35.2	NR	29.8/30.5	NR	16.6/15.8	NR	35.1/34.1
Reference vessel diameter (mm)	2.7/2.6	2.7/2.6	2.7/2.6	3.2/3.0	2.9/2.8/2.8	2.9/2.8/2.9	2.9/2.9	NR	2.8/2.8	NR	2.9/2.8	NR	2.7/2.8
Pre MLD (mm)	NR	0.8/0.7	NR	1.1/1.0	1.1/1.0/1.0	0.8/0.8/0.8	0.8/0.8	0.29/0.51	1.0/0.9	NR	1.2/1.0	1.9/1.9	NR
Post MLD (mm)	3.0/2.9	2.6/2.4	2.6/2.6	2.9/2.9	NR	3.3/3.3/3.3	2.6/2.6	2.8/2.9	2.6/2.5	NR	2.9/2.7	3.4/3.4	NR
Pre-DS (%)	100/100	71.6/75.5	100/100	82.3/79.2	63.3/64.0/65.4	71.4/73.0/71.3	71.1/71.4	92/87	NR	NR	NR	NR	NR
Post DS (%)	7.5/8.2	13.9/15.5	9.0/10.2	14.6/15.3	NR	NR	12.8/13.7	12/12	NR	NR	NR	NR	NR
Multi-vessel disease (%)	49/57	NR	72/63	60/54	NR	NR	68/70	12/9	38/41	83/85	45/45	93/84	53/57
LAD	NR	53/49	NR	56/54	50/52/57	22/19/20	65/60	39/32	64/65	56/53	60/49	NR	48/47
LCX	NR	NR	NR	NR	27/27/20	10/12/7	15/14	16/12	14/14	44/50	18/22	NR	17/17
RCA	NR	NR	NR	29/24	22/22/23	19/20/26	21/25	48/52	22/20	62/58	22/30	NR	25/28
A	NR	NR	NR	NR	NR	NR	NR	NR	NR	NR	NR	NR	NR
B1	NR	NR	NR	NR	NR	NR	NR	NR	NR	NR	NR	NR	NR
B2	NR	NR	NR	73/76	NR	NR	NR	NR	NR	NR	NR	NR	66/68
C	NR	NR	NR	27/24	NR	NR	NR	NR	NR	NR	NR	NR	

*MLD* minimal lumen diameter; *DS* diameter stenosis; *LAD* left anterior descending artery; *LCX* left circumflex artery; *RCA* right coronary artery; *ACC/AHA* American College of Cardiology/American Heart Association; *NR* not reported

### Assessment of quality and publication bias

The risk of bias assessment showed that the selection, attrition, reporting, performance, detection and others bias vary from low to high  (Additional file 1: Figure S1). The funnel plot showed that the distribution was symmetrical for all outcomes (Additional file 1: Figure S2). In addition, the *P-*value of TLR, TVR, MI, MACE, all-cause death, and ST were more than 0.05 by Egger’s and Bgge’s test, which meaned that there were no publication bias. While, the *P-*value of cardiac death outcome by Egger’s test was 0.00 (*P* < 0.05) which implied publication bias (Additional file 2: Table S2). No signs of publication bias was found by the trim method (no new trials added). The quality of GRADE evidence was high for the TLR, MI, MACE, and ST while the quality of evidence was moderate for TVR, cardiac death and all-cause death outcome (Additional file 2: Table S3).

### Trial sequential analysis

Trial sequential analysis (TSA) was performed for each outcome. The cumulative Z curve of TLR and TVR exceeded the traditional boundary and the TSA boundary. Meanwhile, the cumulative Z curve of MACE and ST exceeded the traditional boundary and reach the required information size. However, the cumulative Z curve of cardiac death reach the traditional boundary, while did not exceed the trial sequential analysis boundary and the required information size. In addition, the graph of MI and all-cause death was neither exceeded the traditional boundary nor the TSA boundary (Additional file 1: Figure S3).

### The primary outcome

**Fig. 2:**
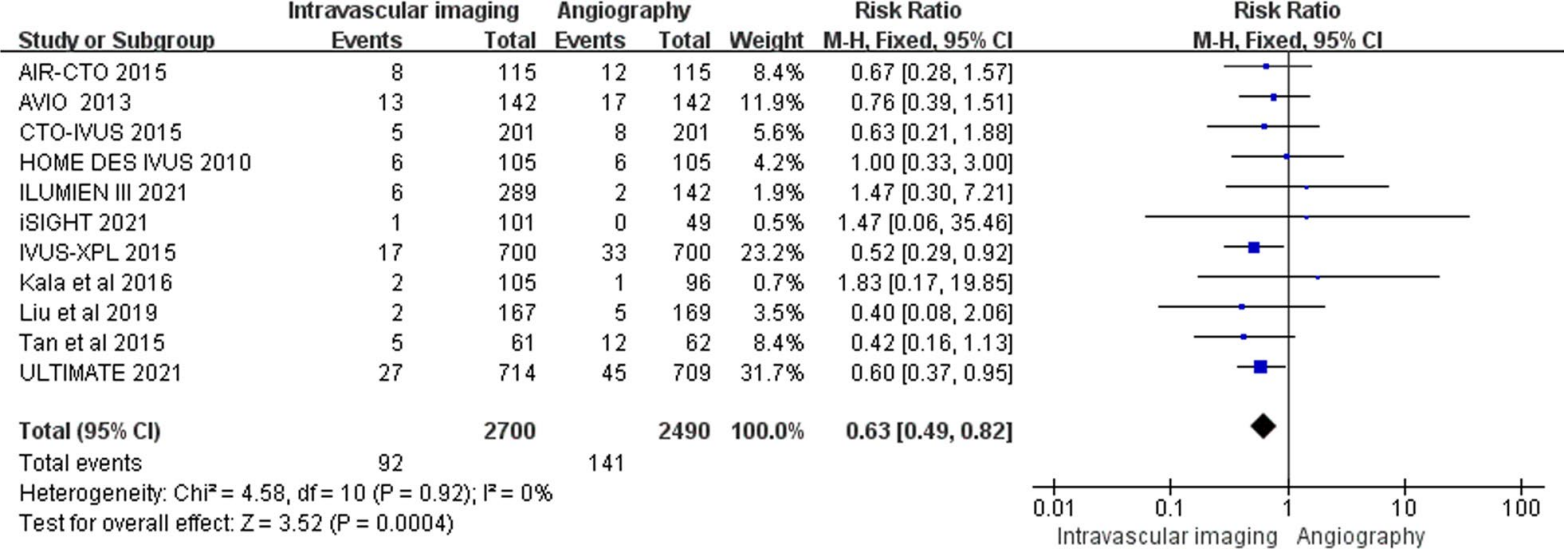
Comparison of the primary outcome between intravascular imaging and angiography guided coronary stent implantation

The risk of TLR was reported in twelve trials (3.4% vs 5.7%, RR 0.63, 0.49–0.82, *P* = 0.0004, *I*^*2*^ = 0%, *P*_*heterogeneity*_ = 0.92), which showed that it is favor of intravascular imaging-guided coronary stent implantation (Fig. 2).

### The secondary outcomes

**Fig. 3:**
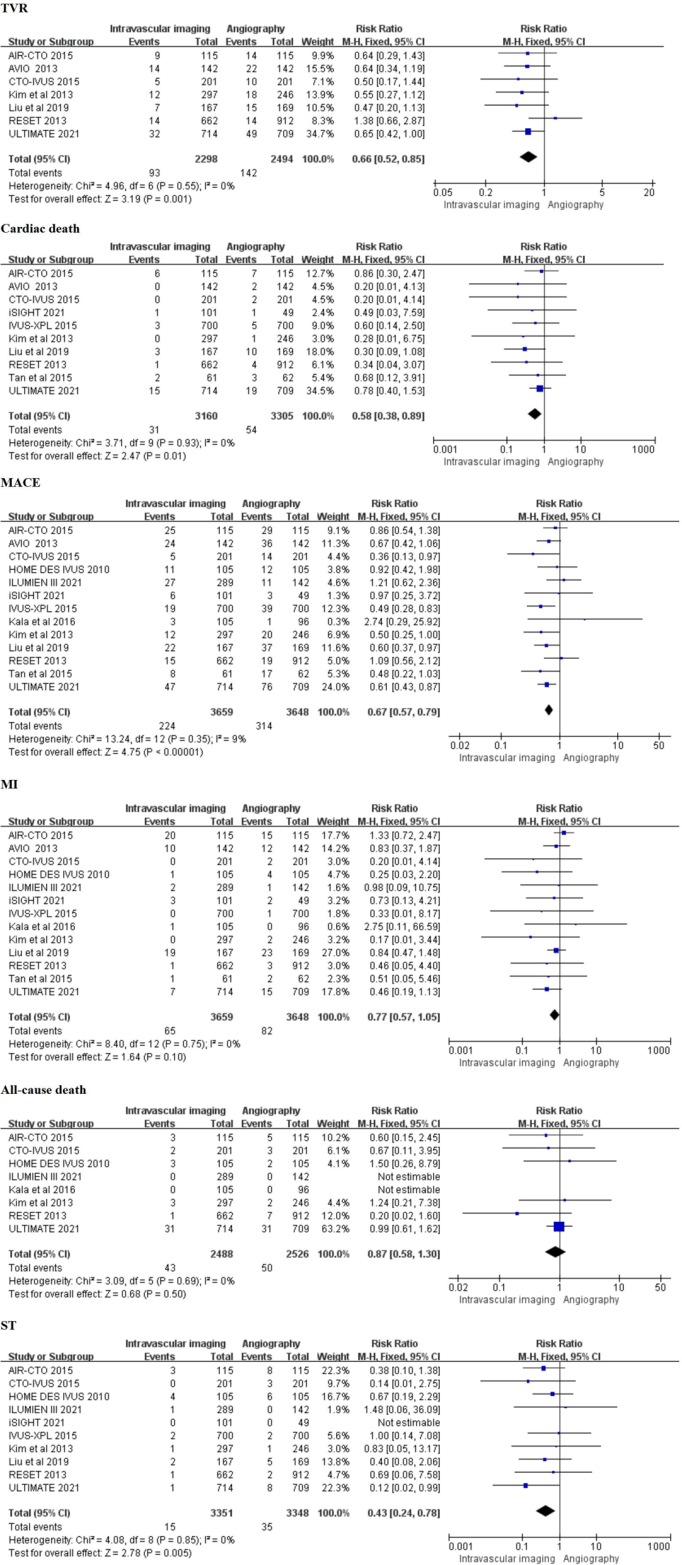
Comparison of the secondary outcome between intravascular imaging and angiography guided coronary stent implantation

The forest map of secondary outcomes was performed (Fig. 3). Of all trials, seven trials reported the event of TVR. The results showed that compared with angiography-guided coronary stent implantation, coronary stent implantation guided by intravascular imaging can significantly reduce the risk of TVR (4.0% vs 5.7%, RR 0.66, 0.52–0.85, *P* = 0.001, *I*^*2*^ = 0%, *P*_*heterogeneity*_ = 0.55). Meanwhile, the cardiac death outcome was established in ten trials, the results demonstrated that the risk of cardiac death was significantly lower in the coronary stent implantation guided by intravascular imaging than that in the angiography-guided coronary stent implantation (0.98% vs 1.6%, RR 0.58; 0.38–0.89; *P* = 0.01) without significant heterogeneity (*I*^*2*^ = 0%; *P*_*heterogeneity*_ = 0.93). The MACE outcome was reported in all trials, and which indicated that intravascular imaging-guided coronary stenting significantly reduced the risk of MACE compared with angiography guidance (6.1% vs 8.6%, RR 0.67, 0.57–0.79; *P* < 0.00001, *I*^*2*^ = 9%; *P*_*heterogeneity*_ = 0.35). In addition, ST was selected as the outcome for eleven trials. The results indicated that intravascular imaging-guided coronary stenting was associated with a reduced risk of ST (0.4% vs 1.0%, RR 0.43, 0.24–0.78; *P* = 0.005) without heterogeneity across the trials (*I*^*2*^ = 0%; *P*_*heterogeneity*_ = 0.85). However, all included trails analyzed the incidence of MI, and eight trials reported the data regarding all-cause death. There was no significant difference in incidence of MI (RR 0.77, 0.57–1.05, *P* = 0.10, *I*^*2*^ = 0%, *P*_*heterogeneity*_ = 0.75), and all-cause death (RR 0.87, 0.58–1.30, *P* = 0.50, *I*^*2*^ = 0%, *P*_*heterogeneity*_ = 0.69) between the two groups.

### Subgroup analyses

Subgroup analyzes were performed according to the lesion types (Additional file 1: Figure S4), presence or absence of left main coronary artery disease (Additional file 1: Figure S5), intravascular imaging type (Additional file 1: Figure S6), and DES type (Additional file 1: Figure S7) to explore the impact of these factors on each outcome. The results showed that intravascular imaging guidance can reduce the risk of MACE (RR 0.62, 0.52–0.73, *P* < 0.00001, *I*^*2*^ = 0%, *P*_*heterogeneity*_ = 0.72) in patients with complex lesions. However, there was no significant difference between the two groups in patients with non-complex lesions (RR 1.14, 0.71–1.84, *P* = 0.58, *I*^*2*^ = 0%, *P*_*heterogeneity*_ = 0.83), and the differences of interaction analysis between the two groups was statistically significant (*I*^*2*^ = 82.7%, *P*_interaction_ = 0.02). In addition, there were no significant differences in the risk of MI (*I*^*2*^ = 0%, *P*_interaction_ = 0.83), all-cause death (*I*^*2*^ = 52.9%, *P*_interaction_ = 0.15) and ST (*I*^*2*^ = 0%, *P*_interaction_ = 0.41) in the subgroup analyzes of lesion types between intravascular imaging or angiography guidance. Furthermore, the results of subgroup analyses according to patients with or without left main coronary artery disease, classification of intravascular imaging, and type of stents showed that no statistical difference was found in relevant outcomes.

## Discussion

This meta-analysis indicates that intravascular imaging-guided DES implantation has a lower risk of TLR, TVR, cardiac death, MACE and ST than coronary angiography-guided DES implantation. Meanwhile, the level of GRADE evidence is high for TLR, MACE, and ST, while the level of evidence of TVR and cardiac death is moderate.

All included studies were randomized controlled trials. However, the risk of bias was high for performance bias owing to only three of thirteen were designed as double blind. Meanwhile, TSA showed that the conclusion of TLR, TVR, MACE, and ST outcomes is reliable and does not need to be verified by more randomized controlled trials. Based on the subgroup analyses, patients with complex lesions seemed to benefit more. The results showed that intravascular imaging guided stenting can reduce the risk of MACE by 38% and ST by 60% in patients with complex lesions. In addition, we performed subgroup analysis of patients with or without left main coronary artery disease, and the results showed that patients with left main coronary artery lesion did not benefit more from intravascular imaging. However, 2018 European Society of Cardiology guidelines on myocardial revascularization suggested that left main coronary artery lesions should be senting guided by intravascular imaging [8]. A meta-analysis of IVUS-guided stent implantation also confirmed that IVUS guidance can improve the clinical prognosis of these patients [25], especially cardiac death, all-cause death, and ST. Considering that sample size was small in this subgroup, the result should be carefully clarified. This meta-analysis searched the basic databases without language restrictions, and the detailed search strategy can be repeated. The Egger’s test showed that cardiac death has publication bias. While the funnel plot has no obvious asymmetry after the trim and fill method.

A meta-analysis of fifteen trials showed that IVUS-guided DES implantation was associated with a significantly reduced risk of MACE in patients with complex lesions in 2017 [26], while this meta- analysis included both randomized controlled trials and observational trials, which may reduce the quality of evidence. Meanwhile, similar to the results of the study, our subgroup analysis also supported that patients with complex lesions have more benefits from MACE. However, there was no heterogeneity between complex lesion and non-complex lesion subgroups in the outcomes of MI, all-cause death, and ST. In addition, in 2019, a study showed that IVUS–guided new-generation DES implantation can reduced the risk of cardiac death, MI, MACE, and ST [27], while the subgroup of our study did not suggest the advantages of new-generation DES compared with first or second generation DES in cardiac death, MI, MACE, and ST outcomes, which may be weakened the clinical benefit of intravascular imaging. Meanwhile, only two studies with new-generation DES were included in our study. Therefore, further large-scale randomized controlled trials are needed to explore it.

The results of this meta-analysis need to be applied cautiously. On the one hand, compared with Caucasians and East Asians, South Asians have a higher incidence of ST-elevation myocardial infarction due to plaque rupture. Meanwhile, the incidence of three-vessel disease and long lesions in South Asians is also significantly higher than that in Caucasians and East Asians [28]. Therefore, South Asians may benefit more from intravascular imaging guidance. However, white and East Asians accounted for the majority of our study, which means it is feasible to guide stent implantation by intravascular imaging in Caucasians and East Asians. In addition, the clinical benefit of intravascular imaging in South Asians needs to be further explored. On the other hand, gender and age may be important factors affecting the nature of plaques. In our meta-analysis, three-quarters of the patients were male nearly, suggesting that intravascular imaging guidance can significantly reduce the incidence of TLR, TVR, cardiac death, MACE, and ST. Meanwhile, some studies show that the plaque burden of patients with male increase significantly with age, and the risk of plaque rupture in patients with male is significantly higher than that in patients with female and the gender difference decreases with age [29–31] and female patients have been benefited more from second generation DES, which inspiring us to further explore the clinical benefits of intravascular imaging-guided DES implantation in different ages and genders. It is reasonable to consider the application of intravascular imaging in patients with male and elderly patients to improve the clinical benefit of subsets.

## Limitation

However, our meta-analysis may have some limitations. Firstly, most of the included randomized controlled trials are small-sample trials, with a low incidence of positive events and wide confidence interval, which reduces the quality of evidence. Secondly, TSA showed that outcome of cardiac death, MI, and all-cause death need more randomized trials are needed to prove it. In addition, the different definitions of MACE and MI in the included trials, which may be one of the reasons for the heterogeneity of MACE outcomes, MI did not get a positive outcome. Meanwhile, MI and MACE was not used as the primary outcome in this meta-analysis. Furthermore, the intravascular imaging included in our study includes IVUS and OCT. Meanwhile, our study included all types of DES, new-generation of DES may lead to better clinical outcomes [32, 33]. However, the subgroup analysis of the first or second-generation and new-generation DES in this study did not get a positive result, which may be related to insufficient sample size and different trials have been associated with different definitions of clinical outcomes. Therefore, further research is needed on the relationship between different DES types and intravascular imaging types. Finally, the underlying disease of patients, the location of lesions, the number of disease vessels, and the specific treatment strategies may also affect the clinical outcome, but our study was a study-level analysis, further analysis cannot be conducted.

## Conclusions

Compared with traditional angiography, DES implantation guided by intravascular imaging can reduce the risk of TLR, TVR, cardiac death, MACE, and ST. In addition, patients with complex lesions will benefit more in MACE. However, whether it is necessary to routinely use intravascular imaging to guide stent implantation still needs to be further explored.

## Supplementary Information

**Additional file 1. Supplementary figure. Figure S1.** Assessment of bias risk for randomized controlled trials included. **Figure S2**. Funnel plot of each outcome. **Figure S3**. Trial sequential analysis for each outcome. **Figure S4**. Subgroup analysis of primary and secondary outcomes between complex lesions and non-complex lesions. **Figure S5**. Subgroup analysis from TLR, Cardiac death, MACE and MI outcomes between left main coronary artery disease and non-left main coronary artery disease groups. **Figure S6**. Subgroup analysis between IVUS and OCT groups. **Figure S7**. Subgroup analysis between first generation and second generation groups.

**Additional file 2. Supplementary table. Table S1.** Search strategy of this meta-analysis. **Table S2**. The *P* value of Begg’s and Egger’s for each outcome. **Table S3**. Summary of GRADE evidence quality for each outcome.

## Data Availability

All data generated or analyzed during this study are included in this published article.
