# Supplementary material for: Efficacy of intravascular imaging-guided drug-eluting stent implantation: a systematic review and meta-analysis of randomized clinical trials
Source: BMC Cardiovasc Disord. 2022 Jul 23;22:327. doi: 10.1186/s12872-022-02772-w (PMC9308935; doi:10.1186/s12872-022-02772-w)
Supplement: Supplementary file 1 — Additional file 1. Supplementary figure. Figure S1. Assessment of bias risk for randomized controlled trials included. Figure S2. Funnel plot of each outcome. Figure S3. Trial sequential analysis for each outcome. Figure S4. Subgroup analysis of primary and secondary outcomes between complex lesions and non-complex lesions. Figure S5. Subgroup analysis from TLR, Cardiac death, MACE and MI outcomes between left main coronary artery disease and non-left main coronary artery disease groups. Figure S6. Subgroup analysis between IVUS and OCT groups. Figure S7. Subgroup analysis between first generation and second generation groups. [file 12872_2022_2772_MOESM1_ESM.docx]

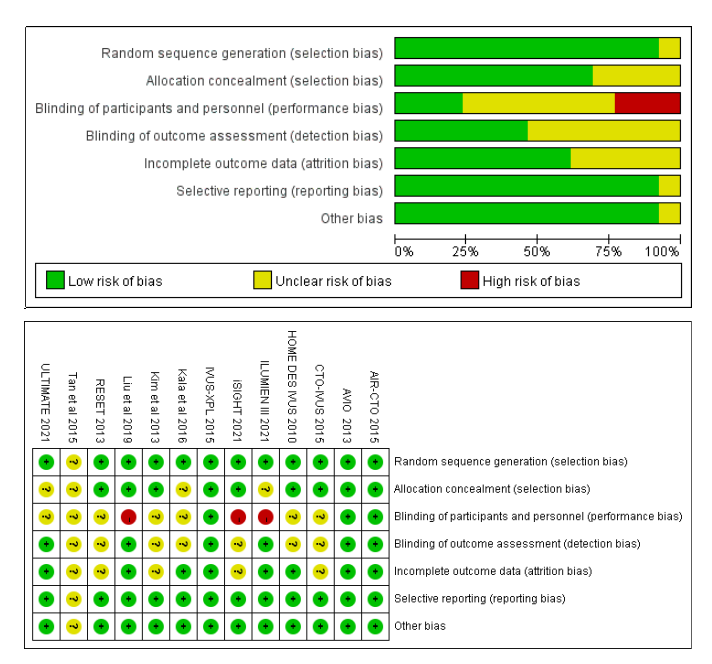


**Figure S1.** Assessment of bias risk for randomized controlled trials included.





**Figure S2.** Funnel plot of each outcome.





**Figure S3.** Trial sequential analysis for each outcome.

**

**

**Figure S4.** Subgroup analysis of primary and secondary outcomes between complex lesions and non-complex lesions.


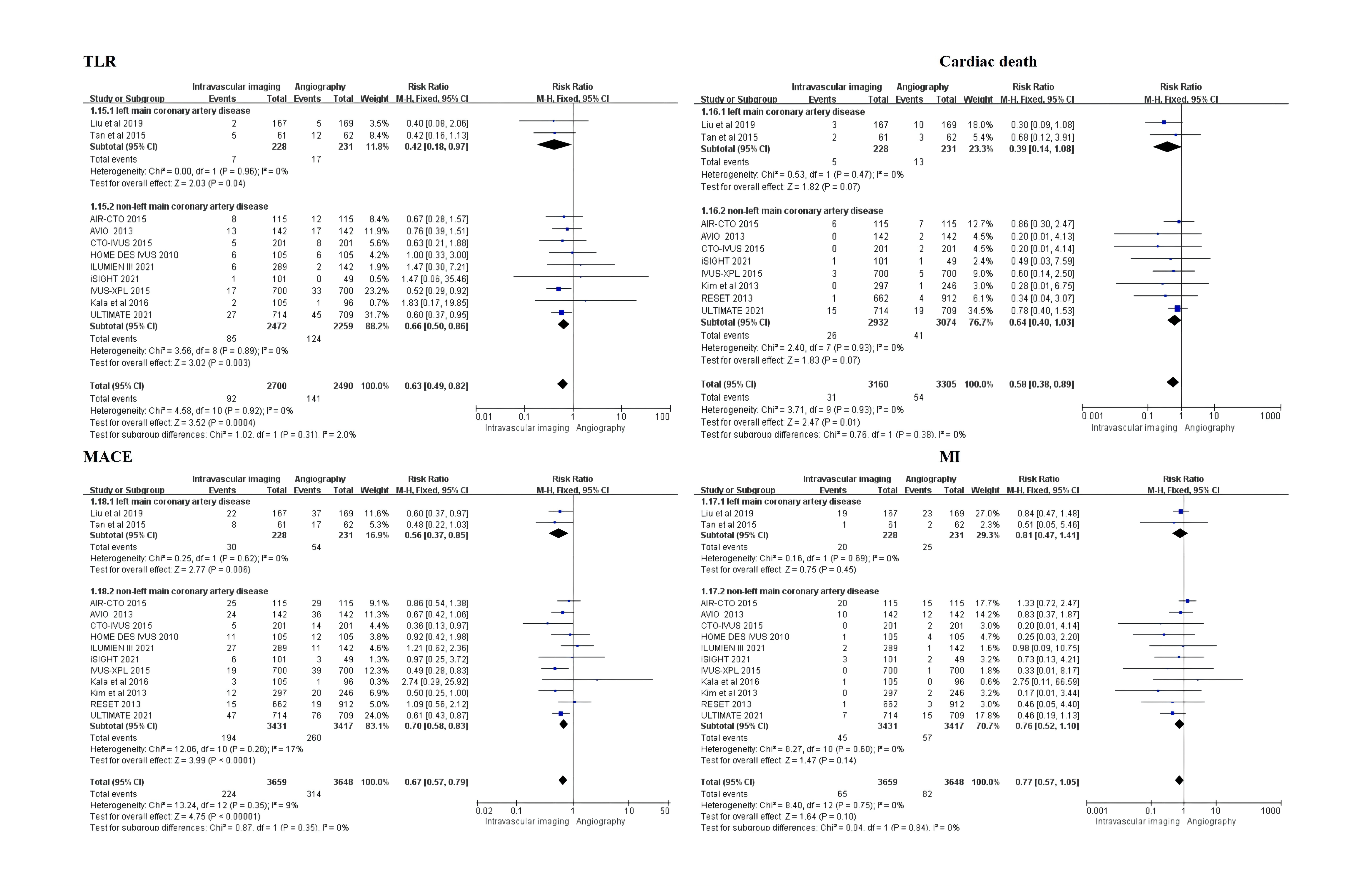


**Figure S5.** Subgroup analysis form TLR, Cardiac death, MACE and MI outcomes between left main coronary artery disease and non-left main coronary artery disease groups.


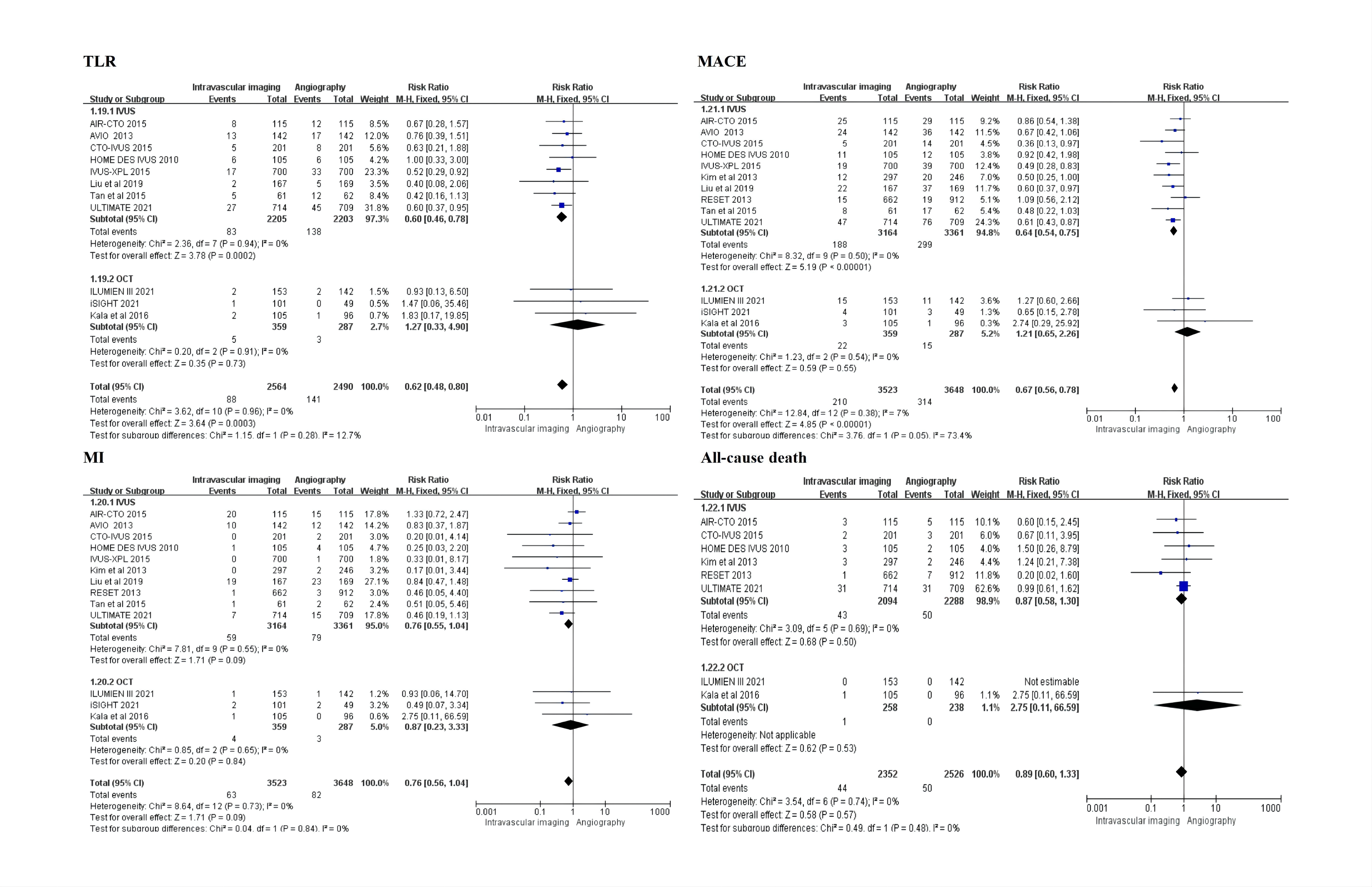


**Figure S6.** Subgroup analysis between IVUS and OCT groups.





**Figure S7.** Subgroup analysis between first generation and second generation groups.
