# Supplementary material for: Efficacy of intravascular imaging-guided drug-eluting stent implantation: a systematic review and meta-analysis of randomized clinical trials
Source: BMC Cardiovasc Disord. 2022 Jul 23;22:327. doi: 10.1186/s12872-022-02772-w (PMC9308935; doi:10.1186/s12872-022-02772-w)
Supplement: Supplementary file 2 — Additional file 2. Supplementary table. Table S1. Search strategy of this meta-analysis. Table S2. The P value of Begg’s and Egger’s for each outcome. Table S3. Summary of GRADE evidence quality for each outcome. [file 12872_2022_2772_MOESM2_ESM.docx]

**Table S1.** Search strategy of this meta-analysis.

| **Searches** | | **Results** |
| --- | --- | --- |
| **Search strategy of Pubmed database.** | |  |
| #5 | Search: (((angiography) OR (angiography guided)) AND (((((intravascular ultrasound) OR (intravascular ultrasound guided)) OR (IVUS)) OR (optical coherence tomography)) OR (optical coherence tomography guided)) OR (OCT))) AND (((stent implantation) OR (percutaneous coronary intervention)) OR (PCI)) Filters: Randomized Controlled Trial | 593 |
| #4 | Search: (((angiography) OR (angiography guided)) AND (((((intravascular ultrasound) OR (intravascular ultrasound guided)) OR (IVUS)) OR (optical coherence tomography)) OR (optical coherence tomography guided)) OR (OCT))) AND (((stent implantation) OR (percutaneous coronary intervention)) OR (PCI)) | 6834 |
| #3 | Search: ((stent implantation) OR (percutaneous coronary intervention)) OR (PCI) | 110293 |
| #2 | Search: ((((intravascular ultrasound) OR (intravascular ultrasound guided)) OR (IVUS)) OR (optical coherence tomography)) OR (optical coherence tomography guided)) OR (OCT) | 3290675 |
| #1 | Seaarch: (angiography) OR (angiography guided) | 336900 |
| **Search strategy of EMBASE database.** | |  |
| #5 | 1# AND #2 AND #3 AND #4 | 529 |
| #4 | 'randomized controlled trial' OR 'randomized' OR rct | 1299857 |
| #3 | 'percutaneous coronary intervention' OR 'stent imlantation' OR pci | 122005 |
| #2 | 'intravascular ultrasound' OR 'intravascular ultrasound guided' OR 'ivus' OR 'optical coherence tomography' OR 'optical coherence tomography guided' OR oct | 791058 |
| #1 | 'angiography guided' OR 'angiography' | 519882 |
| **Search strategy of Cochrane Library database.** | |  |
| #1 | #1 (angiography):ti,ab,kw OR (angiography-guided):ti,ab,kw (Word variations have been searched) | 16755 |
| #2 | MeSH descriptor: [Angiography] explode all trees | 7573 |
| #3 | (intravascular ultrasound guided):ti,ab,kw OR (intravascular ultrasound):ti,ab,kw OR (IVUS):ti,ab,kw (Word variations have been searched) | 1692 |
| #4 | (optical coherence tomography):ti,ab,kw OR (optical coherence tomography-guided):ti,ab,kw OR (OCT):ti,ab,kw | 9198 |
| #5 | MeSH descriptor: [Tomography, Optical Coherence] explode all trees | 1582 |
| #6 | #1 OR #2 | 17427 |
| #7 | #4 OR #5 | 9198 |
| #8 | #3 OR #7 | 10677 |
| #9 | #6 AND #8 | 2070 |
| #10 | (stent imlantation):ti,ab,kw OR (percutaneous coronary intervention):ti,ab,kw OR (PCI):ti,ab,kw | 13986 |
| #11 | MeSH descriptor: [Percutaneous Coronary Intervention] explode all trees | 5993 |
| #12 | #10 OR #11 | 15828 |
| #13 | #9 AND #12 | 471 |
| #14 | (randomized):ti,ab,kw OR (Randomized Controlled Trial):ti,ab,kw OR (RCT):ti,ab,kw | 991421 |
| #15 | #13 OR #14 | 350 |
| **Search strategy of Web of science database.** | |  |
| #5 | #4 AND #3 AND #2 AND #1 | 238 |
| #4 | TS= (randomized controlled trial OR randomized OR RCT) | 98618 |
| #3 | TS= (percutaneous coronary intervention OR stent imlantation OR PCI) | 106570 |
| #2 | TS= (intravascular ultrasound OR intravascular ultrasound guided OR IVUS OR optical coherence tomography OR optical coherence tomography guided OR OCT) | 77873 |
| #1 | TS= (angiography guided OR angiography) | 168124 |

**Table S2.** Summary of GRADE evidence quality for each outcome.

| **Efficacy of intravascular imaging-guided stent implantation:A systematic review and meta-analysis of randomized clinical trials** | | | | | | |
| --- | --- | --- | --- | --- | --- | --- |
| **Patient or population:** patients with stent implantation **Settings:**  **Intervention:** Intravascular imaging VS Angiography | | | | | | |
| **Outcomes** | **Illustrative comparative risks* (95% CI)** | | **Relative effect (95% CI)** | **No of Participants (studies)** | **Quality of the evidence (GRADE)** | **Comments** |
|  | Assumed risk | Corresponding risk |  |  |  |  |
|  | **Control** | Intravascular imaging VS Angiography |  |  |  |  |
| **TLR** Follow-up: 9-36 months | **Study population** | | **RR 0.63**  (0.49 to 0.82) | 5190 (11 studies) | ⊕⊕⊕⊕ **high**^1,2^ |  |
|  | **57 per 1000** | **36 per 1000** (28 to 46) |  |  |  |  |
|  | **Moderate** | |  |  |  |  |
|  | **47 per 1000** | **30 per 1000** (23 to 39) |  |  |  |  |
| **TVR** Follow-up: 9-36 months | **Study population** | | **RR 0.66**  (0.52 to 0.85) | 4792 (7 studies) | ⊕⊕⊕⊝ **moderate**^1^ |  |
|  | **57 per 1000** | **38 per 1000** (30 to 48) |  |  |  |  |
|  | **Moderate** | |  |  |  |  |
|  | **73 per 1000** | **48 per 1000** (38 to 62) |  |  |  |  |
| **MI** Follow-up: 9-36 months | **Study population** | | **RR 0.77**  (0.57 to 1.05) | 7307 (13 studies) | ⊕⊕⊕⊕ **high**^1,3^ |  |
|  | **22 per 1000** | **17 per 1000** (13 to 24) |  |  |  |  |
|  | **Moderate** | |  |  |  |  |
|  | **21 per 1000** | **16 per 1000** (12 to 22) |  |  |  |  |
| **MACE** Follow-up: 9-36 months | **Study population** | | **RR 0.67**  (0.57 to 0.79) | 7307 (13 studies) | ⊕⊕⊕⊕ **high**^1^ |  |
|  | **86 per 1000** | **58 per 1000** (49 to 68) |  |  |  |  |
|  | **Moderate** | |  |  |  |  |
|  | **81 per 1000** | **54 per 1000** (46 to 64) |  |  |  |  |
| **Cardiac death** Follow-up: 9-36 months | **Study population** | | **RR 0.58**  (0.38 to 0.89) | 6465 (10 studies) | ⊕⊕⊕⊝ **moderate**^1,3,4^ |  |
|  | **16 per 1000** | **9 per 1000** (6 to 15) |  |  |  |  |
|  | **Moderate** | |  |  |  |  |
|  | **17 per 1000** | **10 per 1000** (6 to 15) |  |  |  |  |
| **All-cause death** Follow-up: 9-36 months | **Study population** | | **RR 0.87**  (0.58 to 1.3) | 5014 (8 studies) | ⊕⊕⊕⊝ **moderate**^1^ |  |
|  | **20 per 1000** | **17 per 1000** (11 to 26) |  |  |  |  |
|  | **Moderate** | |  |  |  |  |
|  | **12 per 1000** | **10 per 1000** (7 to 16) |  |  |  |  |
| **ST** Follow-up: 9-36 months | **Study population** | | **RR 0.43**  (0.24 to 0.78) | 6699 (10 studies) | ⊕⊕⊕⊕ **high**^1,5^ |  |
|  | **10 per 1000** | **4 per 1000** (3 to 8) |  |  |  |  |
|  | **Moderate** | |  |  |  |  |
|  | **8 per 1000** | **3 per 1000** (2 to 6) |  |  |  |  |
| *The basis for the **assumed risk** (e.g. the median control group risk across studies) is provided in footnotes. The **corresponding risk** (and its 95% confidence interval) is based on the assumed risk in the comparison group and the **relative effect** of the intervention (and its 95% CI).  **CI:** Confidence interval; **RR:** Risk ratio; | | | | | | |
| GRADE Working Group grades of evidence **High quality:** Further research is very unlikely to change our confidence in the estimate of effect.  **Moderate quality:** Further research is likely to have an important impact on our confidence in the estimate of effect and may change the estimate. **Low quality:** Further research is very likely to have an important impact on our confidence in the estimate of effect and is likely to change the estimate. **Very low quality:** We are very uncertain about the estimate. | | | | | | |
| ^1^ Total number of events is less than 300 ^2^ The RR of two studies was less than 0.5 ^3^ The RR of six studies was less than 0.5 ^4^ Egger's test P < 0.05 ^5^ The RR of three studies was less than 0.5 | | | | | | |

**Table S3.** The *P* value of Begg’s and Egger’s for each outcome.

|  | *P* value of Begg’s test | *P* value of Egger’s test |
| --- | --- | --- |
| TLR | 0.117 | 0.276 |
| TVR | 0.548 | 0.817 |
| MI | 0.502 | 0.068 |
| MACE | 0.951 | 0.397 |
| Cardiac death | 0.107 | 0.015 |
| All-cause death | 0.260 | 0.382 |
| ST | 0.917 | 0.989 |
